# Complete mitochondrial genome of *Melia azedarach* L., reveals two conformations generated by the repeat sequence mediated recombination

**DOI:** 10.1186/s12870-024-05319-7

**Published:** 2024-07-08

**Authors:** Zhigang Hao, Zhiping Zhang, Juan Jiang, Lei Pan, Jinan Zhang, Xiufen Cui, Yingbin Li, Jianqiang Li, Laixin Luo

**Affiliations:** 1 Sanya Institute of China Agricultural University, Sanya, Hainan 572025 China; 2 Department of Plant Pathology, Beijing Key Laboratory of Seed Disease Testing and Control, MOA Key Lab of Pest Monitoring and Green Management, China Agricultural University, Beijing, 100193 China; 3 Hainan Seed Industry Laboratory, Sanya, Hainan 572025 China; 4 Department of Pesticide Science, College of Plant Protection, State Key Laboratory for Conservation and Utilization of Bio-Resource in Yunnan, Yunnan Agricultural University, Kunming, Yunnan 650201 China; 5 CAIQ Center for Biosafety in Sanya, Sanya, Hainan 572000 China; 6 Key Laboratory of Vegetable Biology of Yunnan Province, College of Landscape and Horticulture, Yunnan Agricultural University, Kunming, Yunnan 650201 China

**Keywords:** *Melia azedarach*, Mitogenome, MTPT, RNA editing, Phylogenetic analysis

## Abstract

*Melia azedarach* is a species of enormous value of pharmaceutical industries. Although the chloroplast genome of *M. azedarach* has been explored, the information of mitochondrial genome (Mt genome) remains surprisingly limited. In this study, we used a hybrid assembly strategy of BGI short-reads and Nanopore long-reads to assemble the Mt genome of *M. azedarach*. The Mt genome of *M. azedarach* is characterized by two circular chromosomes with 350,142 bp and 290,387 bp in length, respectively, which encodes 35 protein-coding genes (PCGs), 23 tRNA genes, and 3 rRNA genes. A pair of direct repeats (R1 and R2) were associated with genome recombination, resulting in two conformations based on the Sanger sequencing and Oxford Nanopore sequencing. Comparative analysis identified 19 homologous fragments between Mt and chloroplast genome, with the longest fragment of 12,142 bp. The phylogenetic analysis based on PCGs were consist with the latest classification of the Angiosperm Phylogeny Group. Notably, a total of 356 potential RNA editing sites were predicted based on 35 PCGs, and the editing events lead to the formation of the stop codon in the *rps*10 gene and the start codons in the *nad*4L and *atp*9 genes, which were verified by PCR amplification and Sanger sequencing. Taken together, the exploration of *M. azedarach* gap-free Mt genome provides a new insight into the evolution research and complex mitogenome architecture.

**Supplementary Information:**

The online version contains supplementary material available at 10.1186/s12870-024-05319-7.

## Introduction

*Melia azedarach* belongs to family Meliaceae, as a species native and widely distributed in China [1, 2]. This species is renowned not only as a traditional Chinese herbal medicine used to repel rounds and antibacterial [3, 4], but also as a “pesticide plant” with a wide range of applications [5]. Extracts from the *M. azedarach* contain lemon compound and exhibit potent biological activity with environmentally friendly and low toxicity to vertebrates [6]. These exceptional qualities have drawn great attention. Recently, the chloroplast genome [7], and an in-depth analysis of the *M. azedarach* genome has been performed [8]. However, the mitochondria of *M. azedarach* is much less explored.

For most seed plants, nuclear genomic information is contributed by both parents. While, the plastid genome is mostly maternal inheritance, a genetic mechanism that eliminates the influence of paternal-related information and reduces the difficulty of genetic research [9]. According to the theory of endosymbiosis, plant plastid genomes originated from endophytic bacteria [10–12]. They have an independent genetic system from the nuclear genome in long-term evolution [13]. Although mitogenomes, like plastidial genomes, are maternally inherited and contain a smaller gene set, there are significant evolutionary differences between these two genomes [14]. In comparison to mitogenomes, plastidial genomes are relatively compact and remarkably conserved. The chloroplast genome is structurally conserved, generally double-stranded, circular, and contains core genes related to photosynthesis [15]. It has been used for plant evolution analysis because of structurally conservation [16]. For example, the chloroplast genome data provide new insights into the phylogeny and evolution of the genus *Epimedium* [17]. Mitochondria are involved in many metabolic processes of energy transfer and degradation [18]. Mitochondria play an integral role in cell growth and cell development, impacting overall plant growth and development [19]. Previous studies have demonstrated a strong relationship between cellular male sterility (CMS) and mitochondrial gene expression [20]. However, information about CMS in *M. azedarach* has not been reported. There are also some open reading frames of unknown functions in the mitochondrial genome, some of which have a very close relationship with CMS [21]. As a plant with great economic value, deep sequencing of mitochondrial genomes is necessary for utilization and genetic research.

The diversity of mitochondrial structure is a very challenge area for the assembly of Mt genomes [22]. As of April 2023, 12,382 chloroplast genomes and 1,301 plastids were stored in NCBI database, while the number of Mt genomes is only 602. Although most reported plant Mt genomes are described as circular, branching does occur [23]. The size of Mt genome is usually 200-2,000 kb [24]. It has been confirmed that plant mitogenomes tend to exhibit higher mutation rates in comparison to nuclear genomes [25]. This phenomenon has been attributed to the absence of robust DNA repair systems [26]. This higher mutation rate contributes to rearrangements, duplications, and the generation of subgenomic configurations within the mitogenome [27]. Moreover, there is a large amount of sequence rearrangement in the Mt genome, which can lead to multiple configurations of the genome, for example, *Scutellaria tsinyunensis* with two conformations [28]. The presence of numerous similar sequences between chloroplast and Mt genome suggests an information exchange between them [28]. Therefore, next-generation sequencing technology (NGS) is far from meeting the requirements of mitochondrial genome sequencing. In addition, RNA editing within the Mt genome is a post-translational modification phenomenon, leading to differences in sequencing sequences and template sequences [29]. Therefore, it is necessary to employ third-generation sequencing technology with Sanger sequencing for mitogenome research.

In this work, we sequenced, assembled and annotated the Mt genome of *M*. *azedarach*, using BGI short-reads and Nanopore long-reads. The repeat sequence mediated homologous recombination by long reads mapping was analyzed and identified through PCR amplification and Sanger sequencing. In order to explore the sequence migration between chloroplast and Mt genome, the same data was used for chloroplast genome assembly. Furthermore, The RNA editing sites obtained from the bioinformatics analysis were verified by experiment.

## Materials and methods

### Sampling, DNA extraction, and sequencing

In order to obtain the chloroplast and Mt genome, fresh leaves of *M. azedarach* were collected from Yicun, Shandong, China. The plant sample was identified by Zhigang Hao. These specimens have been deposited in our lab (Sanya Institute of China Agricultural University with the accession number SEBIO-2023-1201 and Yunnan Agricultural University with the accession number YNBIO-2023-1221). The total genomic DNA was extracted using a Blood and Cell Culture DNA Midi Kit (Cat. No.13,343, Qiagen, New York, NY, USA) following the manufacturer’s instructions [30]. The extracted DNA was used to construct DNA library with an insert size of 200–400 bp using the BGISEQ platform (BGI, Shenzhen, China). A total of 20 G raw data was produced by DNBSQ sequencing. The plant leaves used for next generate sequence were also used for Oxford Nanopore sequencing. The extracted DNA was prepared for long-read sequencing following the standard processes in the SQK-LSK109 genomic sequencing kit (ONT, Oxford, UK). A total of 10 Gb of sequencing reads was gained via the ONT platform.

### Genome assembly

Firstly, long-reads data was applied for the *M. azedarach* Mt genome assembly with the default parameters using Flye software (version 2.9.1) and the graphical results in GFA format were obtained [31]. The contigs obtained were analyzed using the BLASTN program [32] to identify contig sequence containing Mt genome based on the conserved plant mitochondrial genes in *Arabidopsis*. The parameters utilized were “-evalue1e-5-outfmt6-max _ hsps10-word _ size7-taskblastn-short”. The GFA file was visualized by Bandage software [33], and the Mt contigs were screened according to the results of BLASTn to obtain the raw Mt genome of the *M. azedarach*. Secondly, the data of long-reads and short-reads were mapped to Mt genome using BWA software (version 0.7.17). Then, the reads mapped to Mt genome were filtered and exported for subsequent mixed assembly [34]. Finally, the Mt genome of *M. azedarach* was assembled using Unicycler software [35] by combining the short reads and long reads [34] and visualized use Banage software (version 0.8.1) [33].

### Genome annotation

To predict the gene structure of Mt genome, the published Mt genomes of *Toona sinensis* (NC_065061.1) and *Toona ciliata* (NC_065060.1) were selected as reference genomes, and the Mt genome were annotated by Geseq software (version 2.03) [36]. The tRNA of Mt genome was annotated by tRNAscan-SE software (version 2.0.11) [37]. The rRNA of Mt genome was annotated with BLASTN (version 2.13.0) [32]. The annotation errors of each Mt gene were manually corrected by Apollo software (version 1.11.8) [38].

### Codon usage and DNA repeat sequence analysis

There are great differences in the utilization rate of genomic codons in different organisms, and this preference is considered to be the result of the gradual formation of relative balance during long-term evolutionary selection. Therefore, Relative Synonymous Condon Usage (RSCU) is usually analyzed in genome analysis. the protein coding sequences of Mt genome were extracted by PhyloSuite software (version 1.1.16) [39], and RSCU value of Mt genome was analyzed by MEGA software (version 7.0) [40].

The MISA software (version 2.1), Tandem Repeats Finder software (version 4.09) and REPuter wetsite were used to identify microsatellite sequences, tandem repeats and scattered repeats with the default parameters, respectively [41–43]. The statistical and distributional results of these repetitive sequences were obtained via Excel and Circos package (version 0.69-9) [44].

### Mitochondrial plastid DNAs (MTPTs) identify and synteny analysis

The chloroplast genome was assembled by GetOrganelle software (version 1.7.7.0) [45] and annotated by CPGAVAS2 software [46]. The homologous fragments were analyzed by using BLASTN software [32], and the results were visualized using Circos package (version 0.69-9) [44].

The Mt genomes of the closely related species *T. sinensis*, *T. ciliata*, *Citrus unshiu*, *C. maxima*, and *C. sinensis* were downloaded from the NCBI database with the accession numbers: NC_065061.1, NC_065060.1, NC_057142.1, NC_057143.1, and NC_037463.1. These sequences were used to analyse the covariance between mitochondria by BLAST software [32]. The pairwise comparison of Mt genomes were obtained, and homologous fragments with a length of more than 500 bp were reserved as conservative collinear blocks [47]. The collinear blocks were visualized by Multipe Synteny plot [48].

### Phylogenetic analysis

According to the species relation, the Mt genomes of the related species were downloaded from the NCBI database for further study. The amino acid sequences of shared genes were extracted using PhyloSuite [39], then applied for multiplex sequence alignment though MAFFT software [49]. The aligned sequences were used to construct phylogenetic analysis using IQ-Tree with bootstrap analysis of 1,000 replicates [50]. The phylogenetic tree was visualized using iTol website [51].

### Identification of RNA-editing

All protein-coding genes in the Mt genome were used as input text files to predict editing sites using Deepred-mt [52]. The results were preserved with probability values greater than 0.9 based on a convolutional neural network model.

### Validation of repeat sequence mediated recombination and RNA-editing sites

Primers were designed based on the upstream and downstream sequences flanking of the repeat regions (Table S1, Fig S1, S2). PCR amplification was conducted using 1 µL DNA, 1 uL 10 µM each of the forward and reverse primers, 13 µL 2 × Taq PCR Master Mix, 10 µL ddH_2_O for PCR with the following conditions: 94 ℃ for 3 min; 35 cycles of 94 ℃ for 30 s, 60 ℃ for 3 s and 72 ℃ for 1 min; and 72 ℃ for 10 min. Then, PCR products with the expected size were further sequenced using the Sanger method.

We designed primers to amplify genomic DNA (gDNA) and complementary DNA (cDNA) to validate the RNA-editing sites. The reaction system and condition of the PCR amplification are described in Table S2. The PCR products with the expected size were further sequenced using Sanger sequence. The result was visualized using SnapGene.

## Results

### General feature of the *M*. *azedarach* Mt genome

**Fig. 1:**
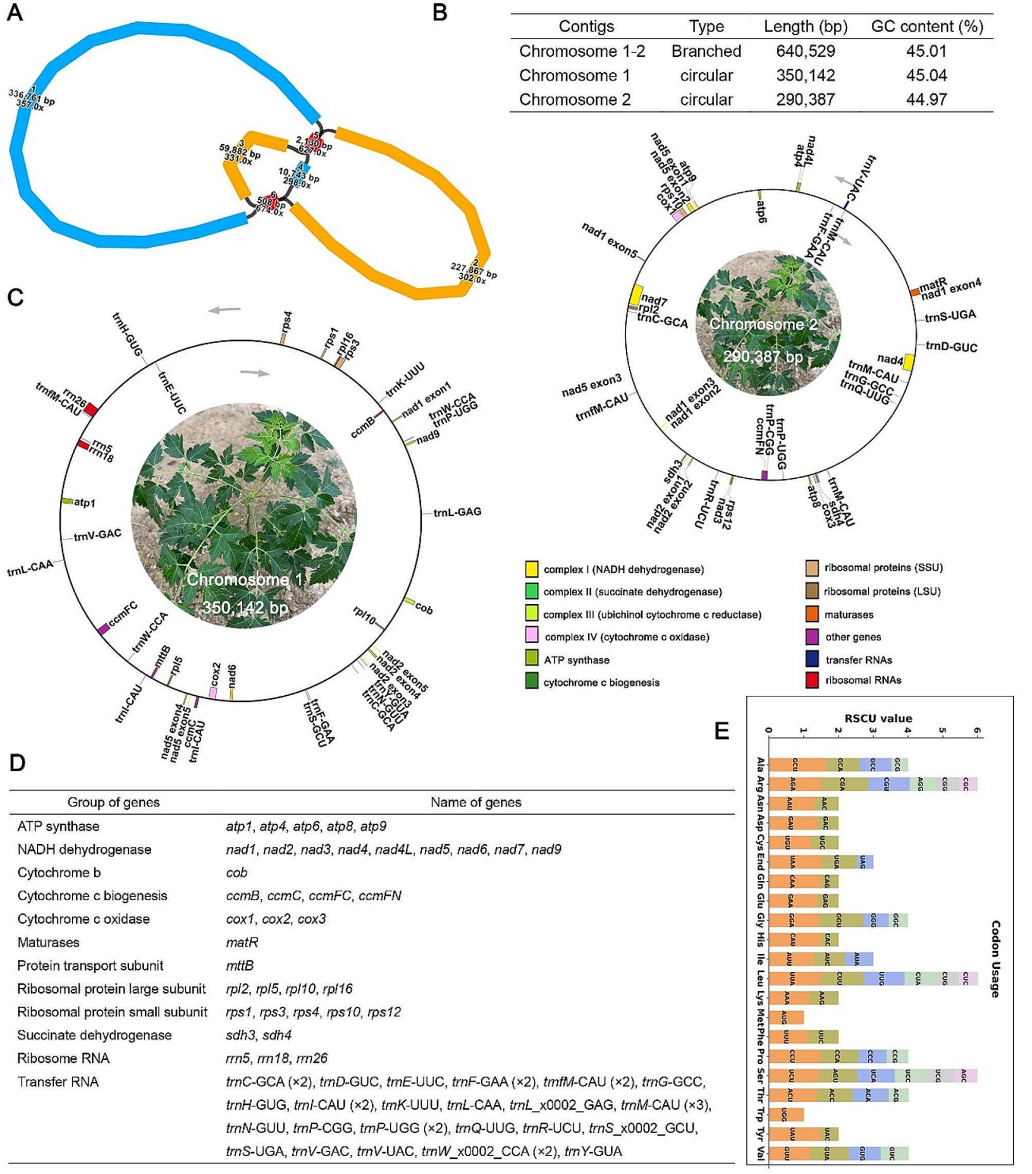
Statistics on *M. azedarach* Mt genomes and their own characteristics. (**A**) The Mt genome of *M. azedarach* in branch. The blue contigs belong to chromosome 1. The yellow contigs belong to chromosome 2. The red contigs belong to both chromosomes. (**B**) Basic Mt genome information including contig number, type, length and GC content. (**C**) Circular maps of the mitogenome. Genomic features mapped on the inside and outside of the circle. Colors were applied for different functional groups. (**D**) Genes predicted in the *M. azedarach* mitogenome. Notes:"2": genes with two copies. (**E**) *M. azedarach* mitogenome relative synonymous codon usage. The codon families are shown on the *X*-axis. The RSCU values are the number of times a particular codon is observed relative to the number of times that codon would be expected for uniform synonymous codon usage

As shown in Fig. 1, A, the Mt genome of *M. azedarach* with graph model was branched, consisting of six contigs. Among them, contig1, contig5, contig4 and contig6 were connected into a circular molecule; contig2, contig5, contig3 and contig6 were connected into a circular molecule. Finally, we obtained two independent circular chromosomes, and the length of chromosomes 1 and 2 was 350,142 bp and 290,387 bp, respectively (Fig. 1, B and C).

The Mt genome was annotated to obtain a total of 35 unique protein-coding genes (PCG), including 24 unique core genes and 11 non-core genes, 23 transfer RNA (tRNA) genes and three ribosomal RNA (rRNA) genes. The core genes include five ATPase genes, nine NADPH deoxygenase genes, four cytochrome C genes, three cytochrome C oxidase genes, one membrane transport protein gene, one mature enzyme gene, and one panthenol-cytochrome C reductase gene. Non-core genes include four ribosomal large subunit genes, five ribosomal small subunits, and two succinate deoxygenase genes (Fig. 1, D).

Codon preference analysis was conducted on 35 PCGs, and the usage of each amino acid codon was shown in Table S3. Codons with relative codon usage (RSCU) greater than 1 are considered to be preferentially used by amino acids. As shown in Fig. 1, in addition to the starting codon AUG and tryptophan (UGG), which both had RSCU values of 1, there was also a common codon usage preference in Mt PCGs (Fig. 1, E). For example, alanine (Ala) has a high usage preference for GCU, with the highest RSCU value of 1.64 among Mt PCGs, followed by arginine (Arg) with a usage preference for AGA, and histidine (His) with a usage preference for CAU, which both have an RSCU value of 1.51. It is worth noting that the maximum RSCU values for phenylalanine (Phe) and valine (Val) were less than 1.2, with no strong codon usage preference.

### Characteristics of repeat sequence

**Fig. 2:**
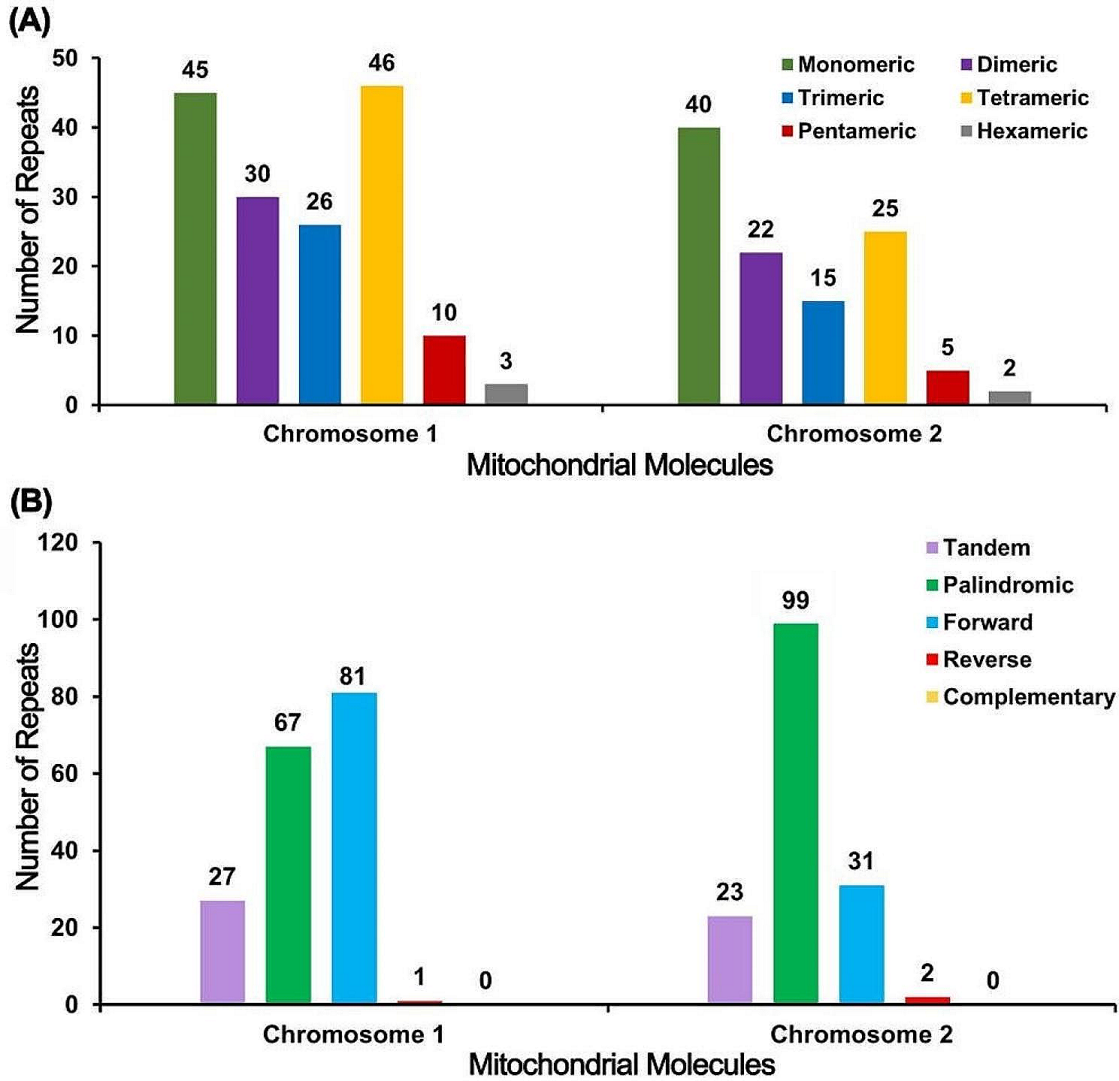
The repeats in *M. azedarach* mitogenome. (**A**) Type and number of SSRs repeats. (**B**) Type and number of tandem and dispersed repeats. Tandem repeats are also known as satellite DNA with multiple copies of repeat units ranging from 7 to 200 bp. As shown in Fig. 2, a total of 27 tandem repeats with the length in 11 ~ 80 bp and the proportion of similarity over 75% was shown in chromosome 1 of *M*. *azedarach*. In chromosome 2, 23 tandem repeats, ranging from 11 to 36 bp were observed (Fig. 2, **B**)

Microsatellite or simple sequence repeats (SSRs) are special type of tandem repeat motif of 1 ~ 6 nucleotides. There were 160,109 SSRs found in *M*. *azedarach* Mt genome. The tetranucleotide polymers accounted for the largest proportion in Chromosome1 with monomeric and dimeric forms of SSRs accounting for 46.88%, adenine (A) monomer repeat accounting for 46.67% (21) of 45 monomer SSRs. Mononucleotide polymers accounted for the largest proportion in Chromosome 2 monomeric and dimeric forms of SSRs accounting for 56.88%, adenine (T) monomer repeat accounted for 52.5% (21) of 40 monomer SSRs (Fig. 2, A).

The dispersed repeats in chromosome 1 and 2 were identified. A total of 149 repetitive sequences (size > = 30 bp) were observed with 81 pairs of forward repetitive sequence and 67 pairs of palindromic repetitive sequence, of which the longest direct repeat and palindromic repeat is 178 bp and 327 bp in chromosome 1. Chromosome2 had 31 pairs of forward repetitive sequence and 99 pairs of palindromic repetitive sequence (Fig. 2, B).

### Repeat sequence mediated the homologous recombination

**Fig. 3:**
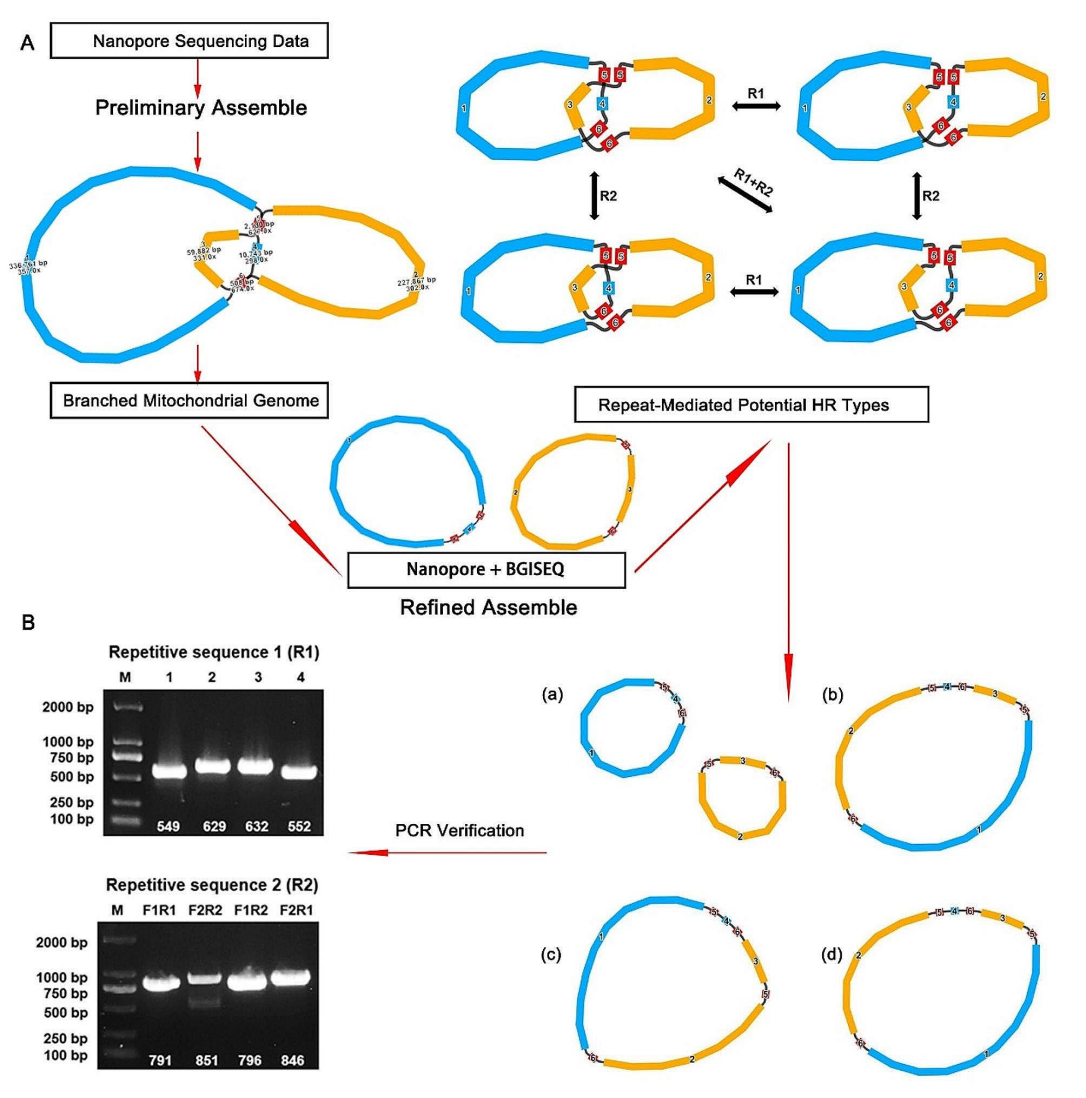
The graphical fragment assembly of *M. azedarach* mitogenome. (**A**) The Mt genome graph and schematic diagram of the HR mediated by different pair repeats (R1, R2). (**B**) The gel electrophoresis results of PCR products amplified using various pairs of primers described in Fig. S1 and Table S1

A lot of studies show that repetitive sequences play an important role in homologous recombination (HR) in Mt genome. Two repeat sequences (R1:contig 5 and R2:contig 6) supported by long reads could be responsible for HR in *M. azedarach* with length of 2,130 bp and 508 bp (Fig. 3, A).

Moreover, to further verify whether the two repeats could mediate HR, PCR amplification and Sanger sequencing were applied. The strategy of the primer was shown in Fig S1 and Fig S2. The primers could amplify different combinations of sequences flanking the repetitive sequences. All PCR products were used for proving the existence of two conformation generated by the repeat-mediated HR (Fig. 3, B), which is consistent with the results obtained by long-reads analysis. The original gel electrophoresis results were showed in Fig S3 and Fig S4.

According to the validation results, we could speculate the potential homologous combination type of the *M. azedarach* Mt genome. The major conformation of *M. azedarach* Mt genome were two circular chromosomes, which could form a separate circular chromosome by R1 and R2. For R1, two conformations can be produced by mediated HR, the other with two small circular molecules and one merged large circular molecule. Similarly, for R2, two conformations can be produced by mediated HR, one with the genome in the structural order contig1-5-4-6-3-5-2-6 and the other with contig1-5-3-6-4-5-2-6. On this basis, repetitive sequences mediate chromosomal recombination to form different configurations.

### Identification of MTPTs

**Fig. 4:**
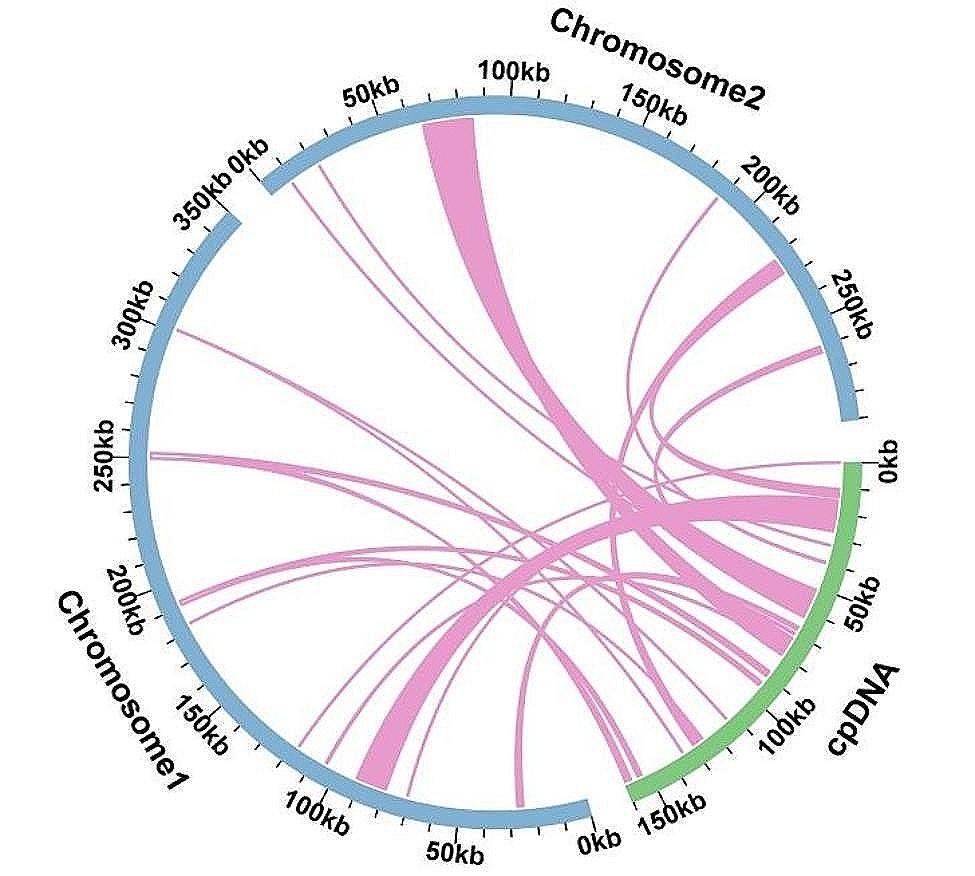
Similar sequences shared between the mitogenome and chloroplast genome. The blue represents the mitogenome. The green represents the chloroplast genome. The purple lines represent homologous fragments

Mitochondrial Plastid DNAs (MTPTs) are fragments of plastid-derived DNA in Mt genomes. Based on the blast analysis for Mt and chloroplast genome, 19 homologous fragments were observed with the length of 46,238 bp, accounting for 7.22% of the total mitogenome (Fig. 4). These MTPTs were distributed across two chromosomes, with 11 MTPTs in chromosome 1, 8 MTPTs in chromosome 2. The longest fragment was MTPT4 with the length was 12,142 bp. There were 36 complete genes in these homologous fragments, including 26 protein coding genes (*acc*D, *atp*A, *atp*B, *atp*E, *atp*I, *clp*P, *ndh*C, *ndh*H, *ndh*J, *ndh*K, *psb*B, *psb*E, *psb*F, *psb*H, *psb*J, *psb*L, *psb*N, *psb*T, *rbc*L, *rpl*2, *rpl* 20, *rpl* 23, *rpo*C1, *rpo*C2, *rps*15, *rps*2) and 10 tRNA genes (*trn*D-GUC, *trn*F-GAA, *trn*H-GUG, *trn*I-CAU, *trn*M-CAU, *trn*N-GUU, *trn*P-UGG, *trn*R-UCU, *trn*V-UAC, *trn*W-CCA) (Table S4).

### Phylogenetic and synteny analysis

**Fig. 5:**
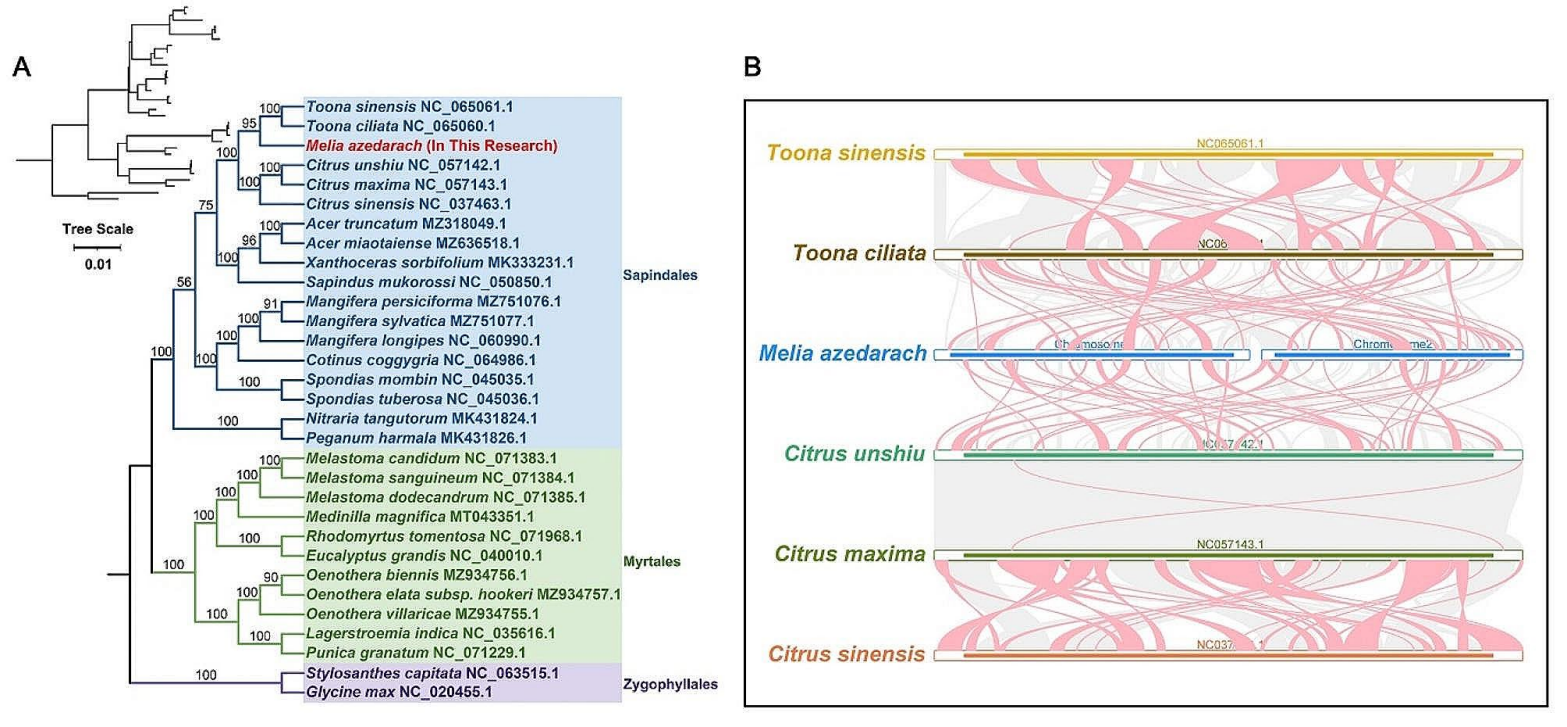
Phylogenetic and synteny analysis of *M. azedarach* mitogenome. (**A**) The phylogenetic relationships of *M. azedarach* with 31 other plants. The colors indicate the families of each species. The left branch represents the phylogenetic relationship constructed by mitochondrial shared genes. The Latin name of each species is followed by the Mt genome NCBI login number. These species are marked on the right. (**B**) *M. azedarach* mitogenomes synteny. Bars indicates the mitogenomes, and the red area means the reversal occurred, the gray areas mean good homology. The white areas mean specific region for species. The blocks (more than 0.5 kb in length) were retained

To realize the evolutionary relationship of *M. azedarach* Mt genome, we constructed a phylogenetic tree using the amino acid sequences of 23 shared Mt PCGs from 31 species (Table S5), namely, *atp*1, *atp*4, *atp*6, *atp*8, *ccm*B, *ccm*C, *ccm*FC, *ccm*FN, *cox*1, *cox*2, *cox*3, *nad*1, *nad*2, *nad*4, *nad*5, *nad*6, *nad*7, *nad*9, *rpl*5, *rpl*16, *rps*3, *rps*14, and *sdh*4. Two species from Zygophyllales were set for outgroups. The topology of the phylogeny consists with the latest classification of the Angiosperm Phylogeny Group. *M. azedarach* belongs to the family Meliaceae and was more closed to *T. ciliata* and *T. sinensis* (Fig. 5, A).

The homologous regions between *M. azedarach* and its closely related species from Sapindales order were identified (Table S6). The highly homologous collinear block was connected by ribbon in this study. As shown in Fig. 5B, the colinear blocks were rearranged in different order. Compared with the closed species, the genomic structure of *M. azedarach* was not conserved. Notably, *Citrus unshiu* and *C. maxima* have good collinearity at the Mt structure level.

### Analysis of RNA editing events

RNA editing events were identified for 35 PCGs form *M. azedarach* using Deepred-mt with cutoff value of 0.9. A total of 356 potential RNA editing sites were observed on 35 PCGs, and all of which were "C to U" base editing (Table S7). The *ccmB* had 37 potential RNA editing sites of the Mt genes, which was the most edited of all mitochondrial genes. This was followed by the *mttB* and *nad7*, each harboring 33 RNA editing events (Fig S5). The vast majority of editing events (93.54%, 333/356) result in amino acid changes, and there were 14 types of amino acid changes, Ser-> Phe, Ser-> Leu, Pro-> Leu, His-> Tyr, Pro-> Ser, Arg-> Trp, Pro-> Phe, Arg-> Cys, Ala-> Val, Leu-> Phe, Arg-> End, Thr-> Ile, Val-> Val, and Thr-> Met.

**Fig. 6:**
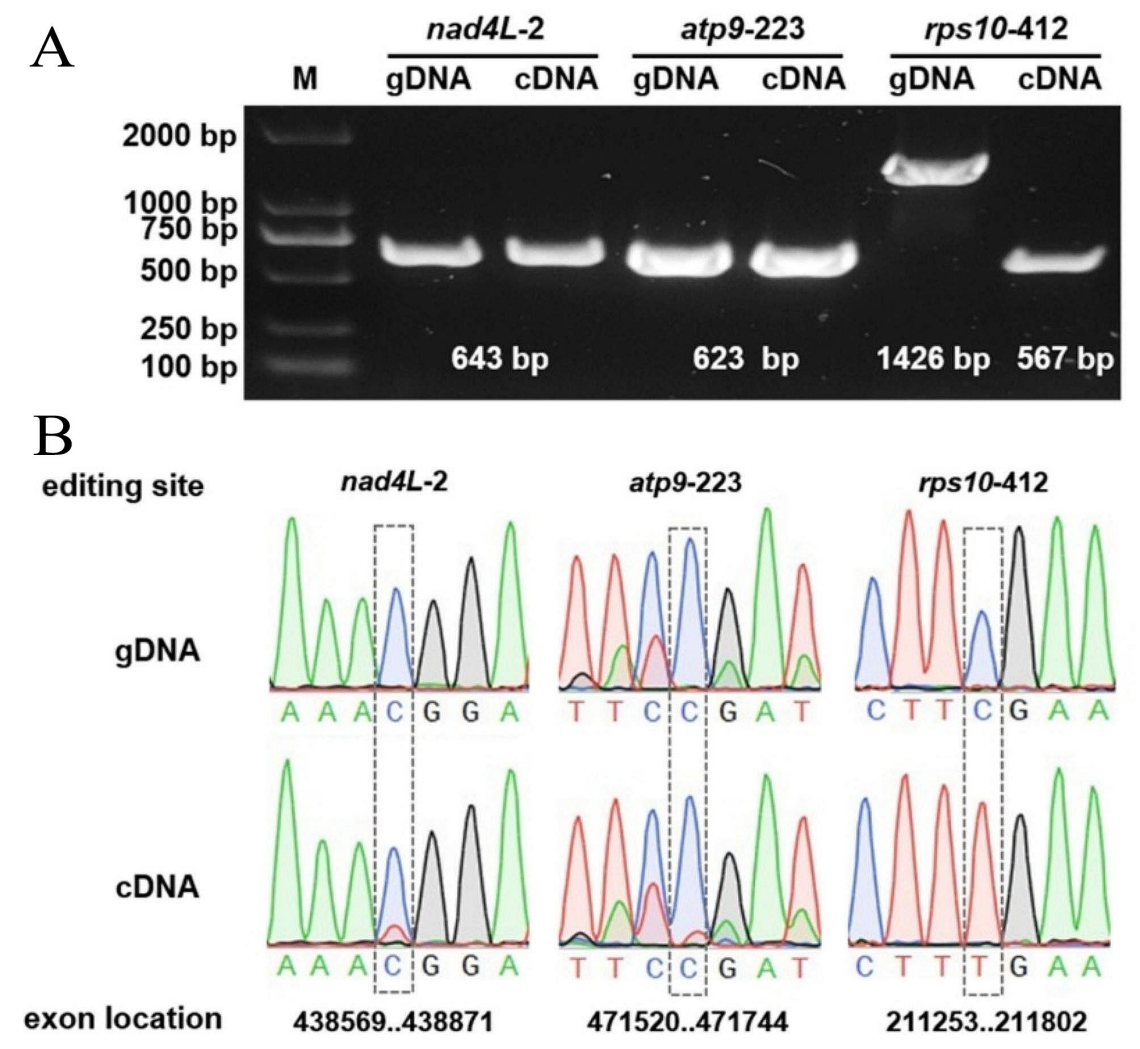
Validation of the RNA events of *M. azedarach* mitochondrial genome. (**A**) PCR verification in the *nad4L*, *atp9* and *rps10* genes of *M. azedarach* mitochondrial genome. (**B**) The gene sequencing results of the gDNA and cDNA. Black dashed rectangles framed the RNA editing sites. The number following the gene represents the site of RNA editing site. The original gel electrophoresis results were showed in Fig S6

Notably, we found that start and stop codons were generated through RNA editing events. Moreover, we validated the existence of these RNA editing events by the PCR amplification and Sanger sequencing. As shown in Fig. 6, the RNA editing sites of *nad4L-2*, *atp9-223* and *rps10-412* were detected. A low-frequency edit had occurred at base 2 of the *nad*4L gene, changing the amino acid sequence from the original Thr to the start codon Met. In contrast, the base 412 of the *rps10* gene has a particularly high editing efficiency among them, changing the amino acid sequence from the original Arg to the stop codon.

## Discussion

Mitochondria are the energy factories of plants, providing essential energy for their growth and development. Here, we described a complete Mt genome of *M. azedarach* with 35 unique genes. *M. azedarach* Mt genome (640,529 bp) has a double circular structure compared to a single circular structure of both *T. sinensis* (638,000 bp) and *T. ciliata* (683,482 bp) [53].

This may be resulted by the number of repeat sequence mediated HR, because the number and proportion of each type of SSR, such as mono-, di-, tri-, tetra-, penta-, and hexa- nucleotide were significantly higher in *M. azedarach* Mt genome, compared to that in *T. sinensis* and *T. ciliate* (Table S8). The configuration of plant mitochondria is more complex than that of animal and fungal. For example, lettuce mitochondria have multiple configurations, including linear, branched, circular structures [23]. Three circular chromosomes (lengths 155, 684 bp, and 45 kb) were existed in cucumber [54]. Therefore, in-depth research on plant mitochondria can enhance our comprehension of the mitochondrial genome evolution and molecular function. Repetitive sequences are widely present in the Mt genome, which is of great significance for the generation of different configurations in mitochondria. Three pairs of repetitive sequences in *Prunus salicina* mediate the production of eight Mt genome configurations [55]. Similarly, there are seven configurations in the Mt genome of *Ipomoea batatas* due to the presence of three pairs of repetitive sequences [13]. In this study, the Mt genome of *M. azedarach* are rich in repetitive sequences, which means they produce multiple configurations in evolution. Two pairs of repetitive sequences, R1 and R2, engender multiple configurations in the Mt genome of *M. azedarach*. We have verified these configurations by PCR amplification and Sanger sequencing. However, their functionality is worth further investigation.

RNA editing is a post-transcriptional modification extensively present in higher plants and constitutes an important step in mitochondrial gene expression, usually related to plant physiology and molecular function [56]. Many studies have shown a correlation between mitochondrial RNA editing and cytoplasmic male sterility [57]. In our study, a total of 356 potential RNA editing sites were identified in 35 unique PCGs, all of which were cytidine-to-uridine (C-to-U) editing. We found that stop codon of the gene (*rps10*) and the start codon of genes (*nad4L* and *atp9*) were created by RNA editing, and verified by PCR amplification and Sanger sequencing. Predicting and identifying these RNA editing sites offers insights into gene functionality predicated on new codons. In the future, it is necessary to further clarify its role in the growth and development of *M. azedarach* by gene editing methods.

A number of plant mitogenomes have acquired genes via horizontal gene transfer from external organisms, incorporating multiple plastid sequences derived from chloroplasts. There has been a transfer of sequences from the plastidial genome to the mitogenome in *M. azedarach* based on sequence similarity (Table S4 and Fig. 4). Analyzing MTPTs sequence helps us to understand the evolution of organelle genome. Based on sequence similarity, a total of 19 shared fragments between mitochondria and chloroplasts were identified, spanning 46,238 bp and accounting for 7.22% of Mt genome length. Among them, MTPT4 was the longest in the length of 12,142 bp. These long MTPTs carry (partial) plastid PCGs to the mitogenome(Table S4). Although partial PCGs frequently evolve into non-functional pseudogenes, they contribute to the diversification of mitochondrial DNA sequences. The tRNA plays a crucial role in sequence migration. A total of 10 tRNA genes have been identified in the migration sequence, indicating that these genes have a significant impact in evolution. Along with some chloroplast fragments entering the mitochondrial genome, mitochondrial DNA sequences become more diverse.

### Electronic supplementary material

Below is the link to the electronic supplementary material.

Supplementary Material 1

Supplementary Material 2

## Data Availability

The mitogenome sequence is available in nucleotide database of GenBank (https://www.ncbi.nlm.nih.gov/nucleotide/) with accession numbers: plastome (PP099858) and mitogenome(chromosome 1: PP099859, chromosome 2: PP099860). The sequencing reads used for mitogenome assembly in this study have been released on the NCBI with those accession numbers: PRJNA1031412 (BioProject); SAMN37977142 (BioSample); SRR26532274 and SRR26532273 (SRA).

## Abbreviations

PCR: Polymerase chain reaction
SSR: Simple sequence repeat
ML: Maximum-likelihood
BI: Bayesian inferences
NCBI: National Center for Biotechnology Information
BLAST: Basic Local Alignment Search Tool
PCGs: Protein-coding gene sequences
MTPT: Mitochondrial plastid DNA
