# Supplementary material for: Complete mitochondrial genome of Melia azedarach L., reveals two conformations generated by the repeat sequence mediated recombination
Source: BMC Plant Biol. 2024 Jul 8;24:645. doi: 10.1186/s12870-024-05319-7 (PMC11229266; doi:10.1186/s12870-024-05319-7)
Supplement: Supplementary file 2 — Supplementary Material 2 [file 12870_2024_5319_MOESM2_ESM.docx]

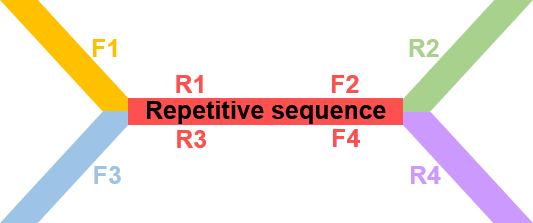


**Figure S1. The primers schematic diagram for validation of the repeat sequence (R1).**


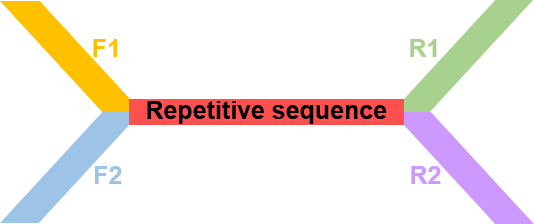


**Figure S2. The primers schematic diagram for validation of the repeat sequence (R2).**

**
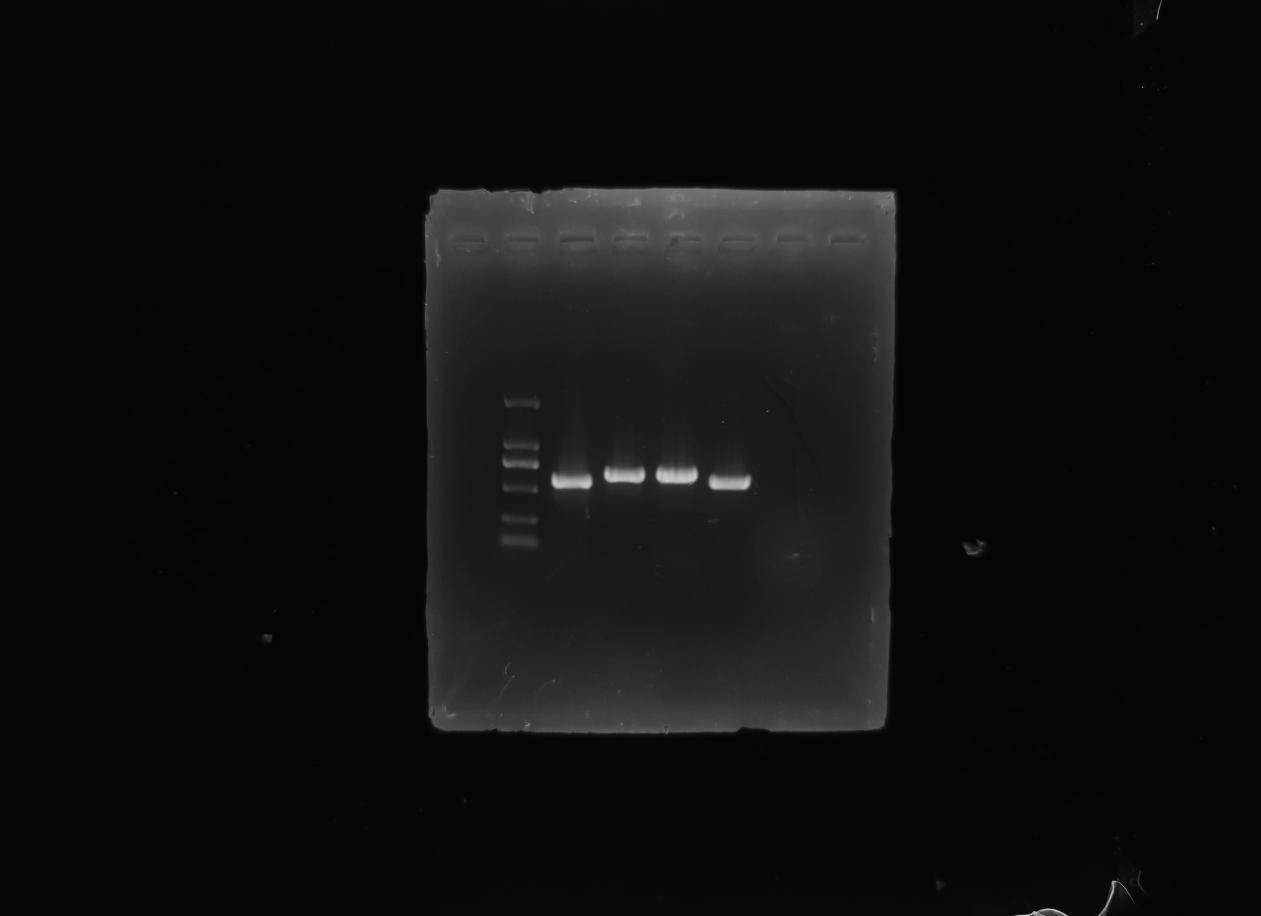
**

**Figure S3. The original gel electrophoresis results of PCR products amplified using various pairs of primers described in Fig. S1 and Table S1.**

**
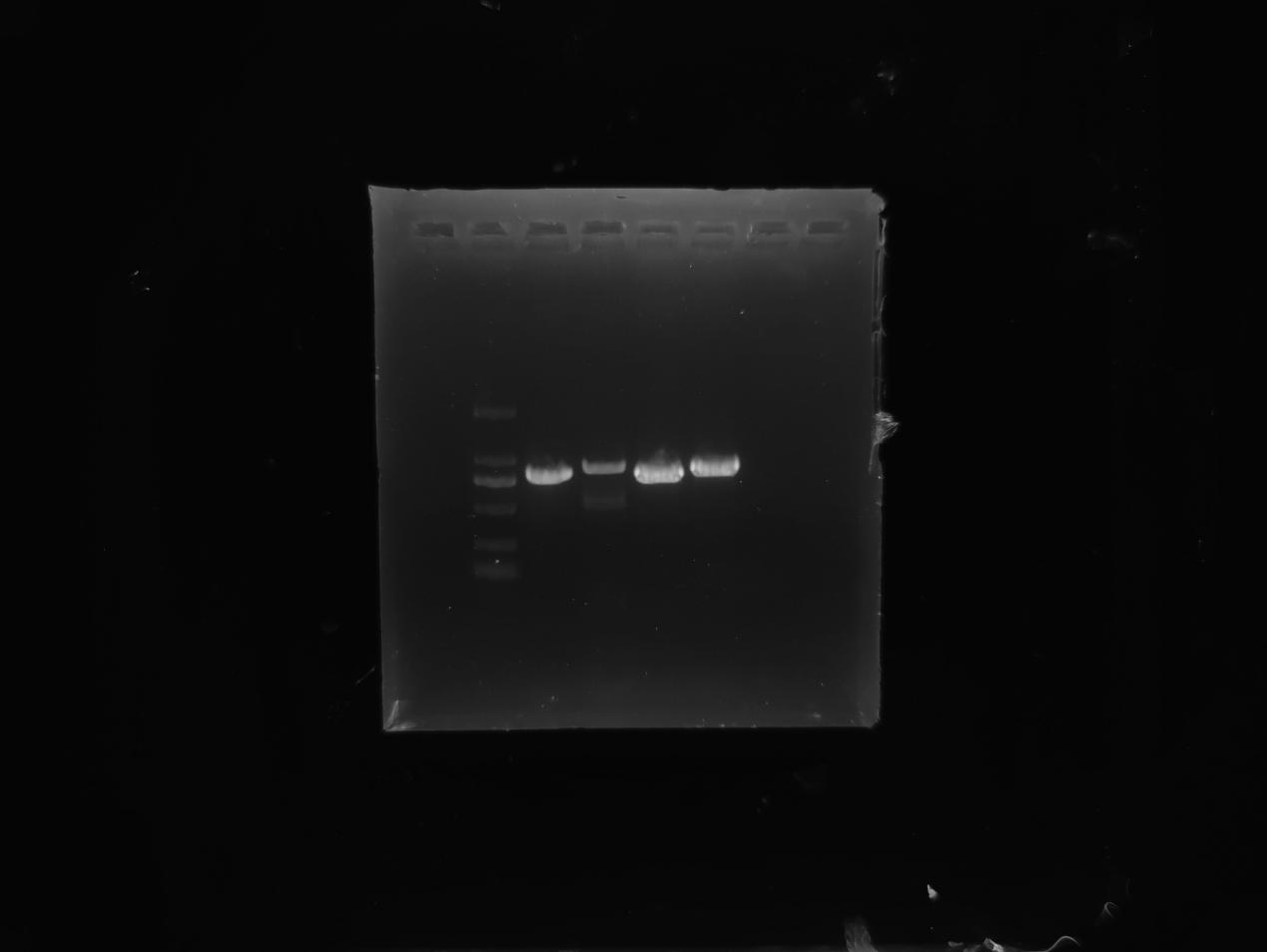
**

**Figure S4. The original gel electrophoresis results of PCR products amplified using various pairs of primers described in Fig. S2 and Table S1.**


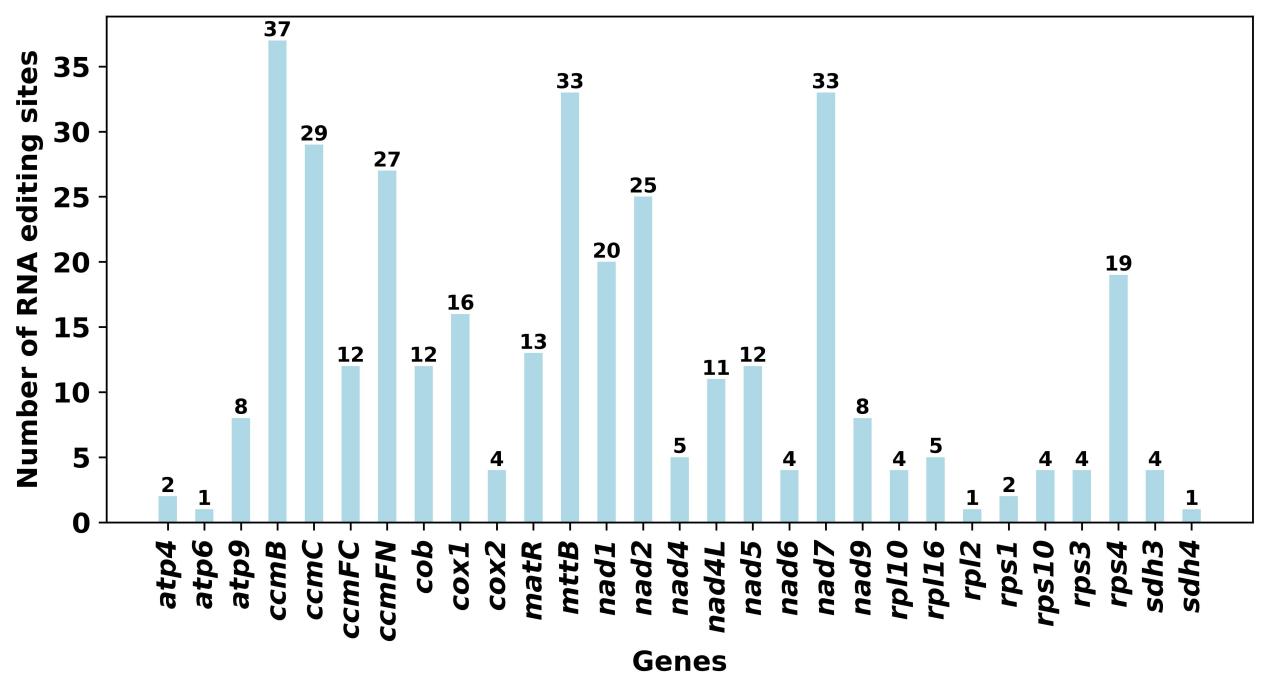


**Figure S5. RNA editing events in *M. azedarach* mitogenome. The *X*-axis shows the gene name. The *Y*-axis indicates the number of RNA edits.**

**
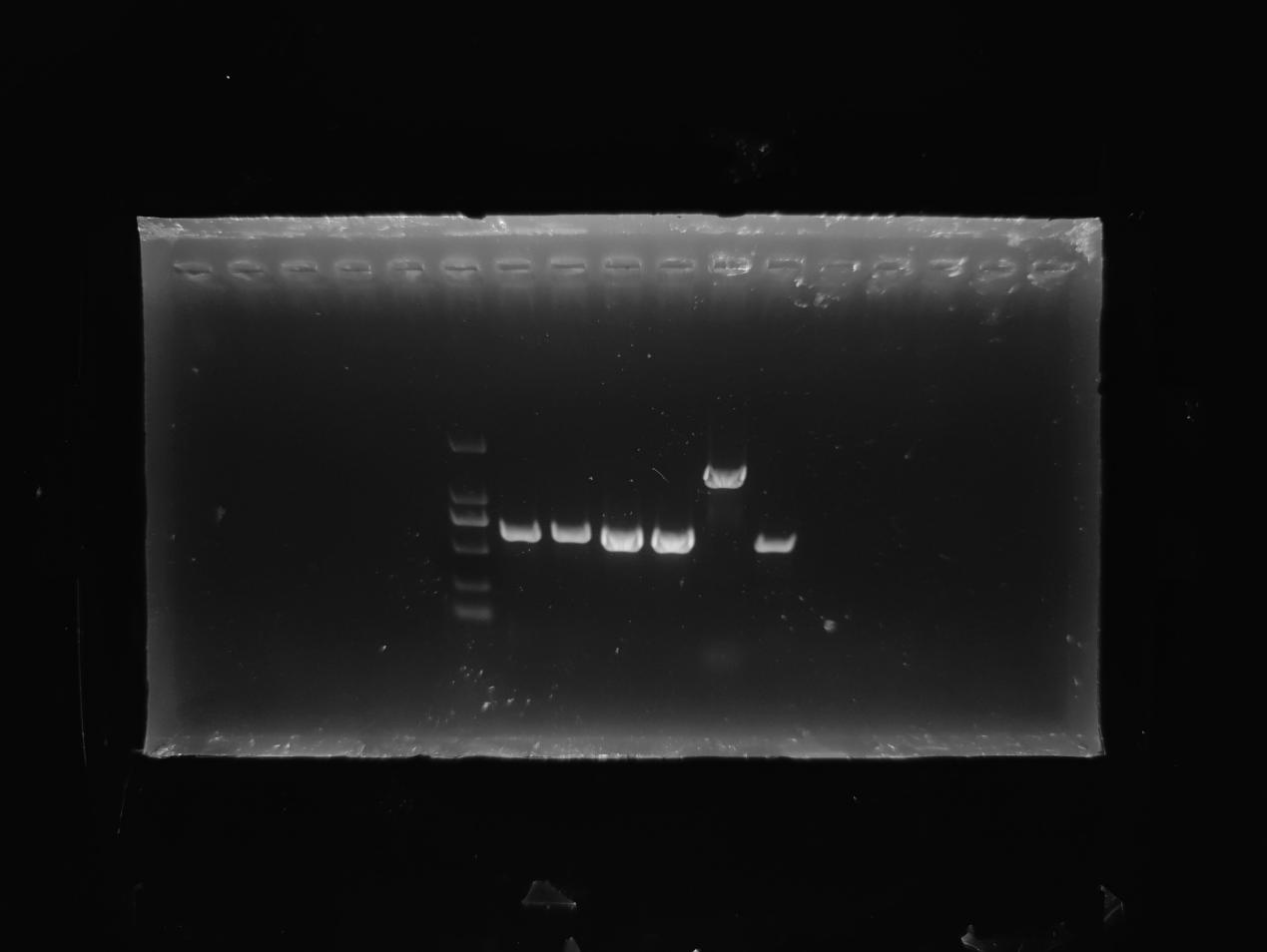
**

**Figure S6. The original gel electrophoresis results of PCR verification for the RNA events in the *nad*4L, *atp*9 and *rps*10 genes.**
